# Reinvigorating the Wiener-Hopf technique in the pursuit of understanding processes and materials

**DOI:** 10.1093/nsr/nwaa225

**Published:** 2020-09-08

**Authors:** David Abrahams, Xun Huang, Anastasia Kisil, Gennady Mishuris, Michael Nieves, Sergei Rogosin, Ilya Spitkovsky

**Affiliations:** Isaac Newton Institute for Mathematical Sciences, UK; College of Engineering, Peking University, China; Department of Mathematics, University of Manchester, UK; Department of Mathematics, Aberystwyth University, UK; School of Computing and Mathematics, Keele University, UK; Faculty of Economics, Belarusian State University, Belarus; Science and Mathematics, New York University Abu Dhabi, UAE

The Wiener-Hopf (WH) method was created in 1931, by Norbert Wiener and Eberhard Hopf, to deliver exact solutions to integral equations with convolution-type kernels on a half-line. It appears that this problem is closely related to that posed by Riemann in 1857 on the problem concerning the construction of a Fuchsian system of differential equations with given singular points and a prescribed monodromy group. This later became known as the 21st Hilbert problem. The WH method is a powerful and long-standing tool that served as a catalyst in broadening the applicability of Fourier analysis, owing to the essential characteristics of analytic functions. Employing the Fourier transform (FT) to the integral equation leads to a Riemann-Hilbert (RH) problem on the real axis:

(1) \begin{eqnarray*} {\Psi ^ + }\!\left( s \right) &+& K\!\left( s \right){\Psi ^ - }\!\left(s \right)= G\!\left(s \right)\!, s \in {\mathbb R},\end{eqnarray*}

where $s$ is the FT parameter, $G$ is a specified function and ${\Psi ^ + },\,\,{\Psi ^ - }$ are unknown functions except that the superscript indicates they are analytic for ${\rm{Im}}( s ) > 0$, Im($s) < 0$, respectively.

An essential component of the method is to *factorize* the given kernel into the form: $K\!( s ) = {K_ + }\!( s ){K_ - }( s ),$ where ${K_ \pm }( s )$ are analytic and non-vanishing functions for $ \pm {\rm{Im}}( s ) > 0$.

Then, multiplying equation (1) by $K_ + ^{ - 1}( s )$ gives

(2) \begin{eqnarray*}\lefteqn{\hspace*{-20pt}K_ + ^{ - 1}\!\left( s \right){\Psi ^ + }\!\left( s \right) + {K_ - }\!\left( s \right){\Psi ^ - }\!\left( s \right)}\nonumber\\ &=& K_ + ^{ - 1}\left( s \right)G\!\left( s \right),\,\,s \in \mathBB{R}.\end{eqnarray*}

Finally, using a combination of Cauchy's integral formula, Liouville's theorem and analytic continuation arguments, explicit evaluation of ${\Psi ^ \pm }( s )$ is enabled. Note that solutions to the homogeneous equation (1) (i.e. $G( s ) = \,\,0$) provide information on the kernel space of the original integral operator.

The general factorization procedure for the scalar RH problem (1) on a closed curve $\Gamma ,\,\,$dividing the complex plane into two domains ${D_ + } \cup \,\,{D_ - } =\, $**${\mathBB C}$**, was established by Gakhov in 1933.

A natural generalization of equation (1) assumes this represents $n$ equations on $\Gamma $, leading to a factorization of a matrix kernel, $K\!( s ),$ via a product of $n \times n$ matrices:

(3) \begin{equation*}K\!\left( s \right) = {K_ + }\!\left( s \right)D\!\left( s \right){K_ - }\!\left( s \right)\!,\quad s \in \Gamma ,\end{equation*}

with $D( s ) = {\rm{dia}}{{\rm{g}}_{1 \le k \le n}}\{ {{s^{{i_k}}}} \}$, where $\{ {{i_k}} \}_{k=1}^n$ are the *partial indices*. Considerable challenges arise here and so far a general approach to obtaining equation (3) remains elusive.

In 1958, two seminal papers appeared: one by Krein, dealing with the scalar equation$,$ and the other, co-authored by Krein and Gohberg,

focusing on the vector case. Both demonstrated direct links between linear operator analysis and the factorization problem.

However, in contrast to the scalar case, a general procedure for constructing equation (3) still remains an open problem. The validity of any numerical approximate factorization is also questionable as they proved (independently of Bojarski, whose proof appeared the same year) that the indices $\{ {{i_k}} \}_{k= 1}^n$ are stable to perturbations if and only if

(4) \begin{equation*}{\rm{max}}\!\left\{ {{i_k}} \right\}_{k=1}^n - {\rm{min}}\!\left\{ {{i_k}} \right\}_{k=1}^n \le 1.\end{equation*}

Since then, the theory has undergone significant development in determining classes of matrix functions allowing for constructive factorization. For analytic and rational matrix-functions, a finite factorization algorithm and the method of pole removal have been developed. Important classes of non-rational matrix-functions and piecewise constant matrices, appearing in RH problems related to the construction of complex differential equations with a prescribed monodromy, were studied. Factorization methods for matrix-functions having special properties (e.g. positive definite, unitary, self-adjoint, circular, triangular) have also been developed. Alternative factorization methods (spectral, commutative, non-commutative etc.) also exist (see [1] and references therein). Methods to approximate matrix factorizations have also been proposed for certain classes of matrix functions including a popular approach using rational approximations of matrix entries (see [2] and references therein).

In particular, while the aforementioned approaches have allowed the WH-RH method to have an immense impact in the solution linear mixed problems it is clear the approach has limitations in solving nonlinear problems. Nevertheless, some nonlinear problems are treatable with these methods, including those containing Painlevé-type equations.

Traditional applications of RH-WH techniques, developed by Muskhelishvili and others, include elasticity, plasticity, fracture mechanics and wave scattering by obstacles with simple geometries. In the last 50 years the method has also provided a profound impact in finance, aerospace, acoustics, electromagnetics, water waves, civil engineering, advanced materials, etc. However, further progress is severely constrained due to the aforementioned issues, as most new applications lead to matrix problems. Nevertheless, problems arising from applications can offer natural pathways to solving WH problems, e.g. wave diffraction by complex surfaces can be reformulated as a sequence of simple approximate WH problems [3]. Additionally, the method can reveal pertinent information about physical problems, e.g. the appearance of leaky waves potentially missed by standard numerical procedures.

**Figure 1.**
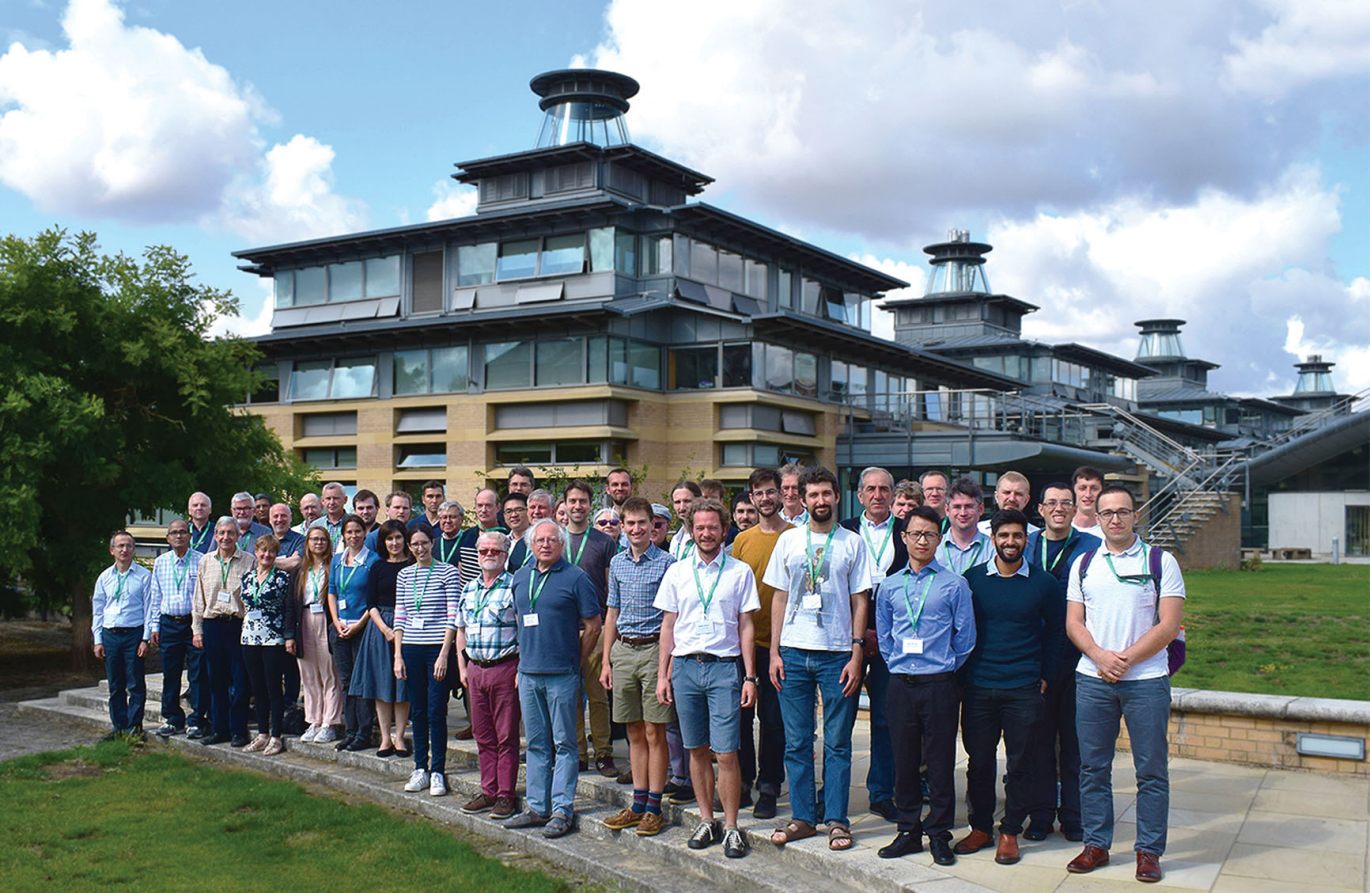
The participants of the WHT research programme held at the Isaac Newton Institute for Mathematical Sciences during 5–30 August 2019.

Driven by a range of novel real-world applications, the recent WHT programme gathered international experts in the WH method (see Fig. 1) to initiate a renewed effort to address existing challenges concerning matrix factorization.

In aerospace applications, matrix factorization techniques have created new possibilities in understanding turbofan noise radiation and its reduction by various edges and soft boundaries. Consequently, this has revealed insight concerning silent flying capabilities of owls [3] that could, amongst other impacts, revolutionize current aircraft designs with environmental benefits.

**Figure 2.**
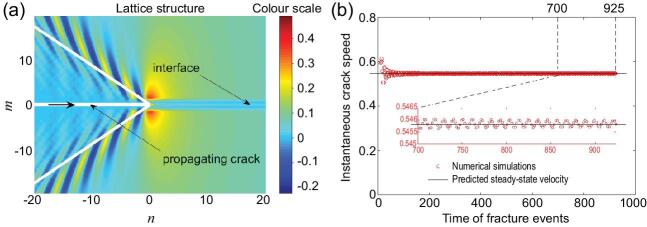
(a) Deformation induced by a subsonic crack (situated at $n < 0,\,\,m = 0$) propagating between two identical isotropic lattices connected by a stiff non-inertial interface (situated at $n > 0,\,\,m = 0$). The interface is composed of links four times stiffer than those in the ambient medium. The crack propagates with a speed that is 90% of the speed of sound in the lattices. The colour plot (scale shown on the right) indicates the change of length in the vertical lattice links relative to their original length, with positive (negative) values representing an extension (compression) of these links. As the crack propagates, energy is released into the medium, with the white inclined lines indicating the most preferable directions for this process. (b) Typical fluctuation in the velocity of a subsonic crack propagating in a uniform discrete structure (see [6] for more details).

A popular driver of new technologies includes metamaterials, possessing unusual static and dynamic properties at multiple scales unavailable in natural materials (e.g. negative Poisson's ratio). Waves propagating through (or redirected by) metamaterials may promote high local stress concentrations, making these materials susceptible to failure. The WH technique has already proven its efficacy for such problems [4]. Figure 2(a) shows a subsonic crack propagating within a discrete uniform lattice along an interface. Note in this case that equation (1), with the right-hand side, $G( s )$, represented by the Dirac delta function, defines all waves radiated from the crack tip.

Metamaterials are natural candidates that require matrix factorization due to their complexity and rich multi-scaled response. Examples include the existence of various failure regimes (steady state, clustering, forerunning [5]), their topological structure and possible behavioural instabilities. Note that in such materials, even steady failure can be difficult to realize. The failure speed is *deterministic and usually oscillates* around the average value as is shown in Fig. 2(b) for the case of uniform discrete structures [6].

The importance of the WH method in bridging and advancing seemingly unrelated fields is illustrated in its application to financially inspired Levi processes or discretely monitored path-dependent option pricing. Other promising directions include the development of hybrid methods, combining analytical modelling, artificial intelligence and experimental testing [7]. Potential benefits include the rapid production of high-fidelity ground truth data for the training of machine learning models and the development of forward models required by inverse testing methods applied in acoustic and ultrasound imaging. The method has also recently opened new directions in analysing wave phenomena in complex solids with random microstructure (see [8] and references therein).

All these challenging problems face issues concerning possible instabilities of the partial indices and, in the absence of the generalized approach, require significant advancement of matrix factorization techniques. Possible promising directions have been recently reported [9,10].

Since its conception, the WH technique has remained an enduring analytical tool used broadly in addressing many challenging real-world problems. Its ubiquity, elegance and insight will ensure its continued importance to researchers in a plethora of fields, creating new mathematical research paradigms and enabling scientists to unlock existing and novel industrial and societal challenges.
